# Genital lesions in cows naturally infected with trypanosomes in Abuja, Nigeria

**DOI:** 10.14202/vetworld.2021.1363-1370

**Published:** 2021-05-29

**Authors:** Kenneth Owoicho Abah, David Ogwu, Lushaikyaa Allam, Christopher Ese Obudu, Joy Iyojo Itodo, Nuhu Abdulazeez Sani

**Affiliations:** 1 Department of Theriogenology, Faculty of Veterinary Medicine, University of Abuja, Nigeria; 2 Department of Theriogenology and Production, Faculty of Veterinary Medicine, Ahmadu Bello University, Nigeria; 3 Department of Animal Science, Faculty of Agriculture, Federal University of Lafia, Nigeria; 4 Department of Veterinary Pathology, Faculty of Veterinary Medicine, University of Abuja, Nigeria

**Keywords:** Abuja, cow, genital organs, lesions, trypanosomosis

## Abstract

**Background and Aim:**

Different species of trypanosomes have been reported to cause varying degrees of reproductive disorders in pregnant and non-pregnant animals under experimental infections. Information on reproductive disorders and losses in animals naturally infected with trypanosome species are few. This study was carried out to assess the abnormalities in the genital organs (ovaries, oviduct, uterus, cervix, and vagina) of female cattle naturally infected with trypanosomes in and around Abuja, Nigeria.

**Materials and Methods:**

Cows showing signs such as emaciation, weakness, or anemia were selected and examined at Gwagwalada and Karu abattoirs, respectively. Venous blood samples were taken from 108 of such animals and screened using standard trypanosome detection methods. The genital organs were also collected and inspected for gross and histopathological lesions in the laboratory.

**Results:**

Six (5.55%) out of the 108 animals were positive for trypanosomes; 4 (66.7%) were infected with *Trypanosoma* *vivax* and 2 (33.3%) were infected with *Trypanosoma congolense*. The mean packed cell volume of the infected animals was 22.83%. Grossly, congestion and ecchymotic hemorrhages were observed in the endometrium, myometrium, and cervical submucosa. Mucometra, hydrometra, and pyometra were also seen in the uterus. Histologically, necrosis of the epithelium and endometrial glands accompanied by mononuclear cellular infiltration was observed in the uterus. There was also sloughing of the endometrial epithelium, vascular congestion, and hypertrophy of serosa of the uterus. There was atropy of the granulosa cells, increased numbers of degenerating tertiary follicles, and absence of corpora lutea in the ovary. No gross or histopathological lesions were observed in the fallopian tube and vagina.

**Conclusion:**

The lesions observed were restricted to the uterus and ovary of the animals and were less severe when compared to lesions observed under experimental conditions as reported by previous authors.

## Introduction

Trypanosomosis is an infectious disease caused by pathogenic blood parasites known as trypanosomes. Trypanosomosis is prevalent over about one-third of the total African land mass. *Trypanosoma* species are transmitted by tsetse flies (*Glossina* spp.) and other biting insects [1,2]. Four species of *Glossina* (*Glossina palpalis* *palpalis*, *Glossina tachinoides*, *Glossina morsitans* *submorsitans*, and *Glossina longipalpis*) are important in the transmission of the disease in Nigeria [2]. Biting insects of the genus *Tabanus*, *Stomoxys*, and *Hippobosca* are also involved in the mechanical transmission of the disease [3,4]. The disease affects both humans (human African trypanosomosis or sleeping sickness) and animals (African animal trypanosomosis or Nagana). Common trypanosome species that infect cattle are *Trypanosoma vivax*, *Trypanosoma Congolense*, and *Trypanosoma brucei* [5]. Clinical signs of African animal trypanosomosis in cattle are weakness, lethargy, weight loss, anemia, lacrimation, fluctuating pyrexia, roughened coat, superficial lymphadenopathy, and sometimes death of the animal [6]. Animal trypanosomosis constitutes a major threat to food security in several parts of sub-Saharan Africa [5,7]. It is estimated that not <46 million cattle are at risk of becoming infected by tsetse-transmitted trypanosomosis [5,7]. Animal trypanosomosis has caused not <3 million livestock deaths, 25% reduction in milk yields, 50% reduction in livestock numbers and has reduced work efficiency of animals, thus hindering crop production [7].

Several studies show that trypanosomosis cause a wide range of reproductive disorders in animals, as well as degeneration of the gonads with consequent disruptions in the secretions and plasma levels of the hormones necessary for normal reproductive processes in both male and female animals [8-15]. Genital abnormalities that have been observed in female animals included anestrus, irregular estrus cycles, low birth weight, stillbirth, neonatal death, abortion, and premature birth [16-19] and were associated with vertical transmission through the placenta [20,21]. Whereas in male animals infected with trypanosomes, the abnormalities that have been observed were severe degenerative changes of the genitalia (testis and epididymis), delayed puberty, reduced libido, orchitis, and poor semen characteristics [22-24]. Sterility, menstrual disorder, and stillbirth have been reported in humans during trypanosomosis infection [25]. Most of these are, however, results of experimental infections.

This study was designed to describe the histopathological lesions in the reproductive organs of cows naturally infected with trypanosomes and to determine the prevalence of trypanosome infected cattle brought for slaughter at the Abuja abattoir.

## Materials and Methods

### Ethical approval

Blood samples were collected without any injury to the animals based on standard sampling procedures. Approval was not sought from the institution’s animal ethics committee because the study did not affect normal animal physiology.

### Study period and location

The study was conducted from April 2018 to August 2018 in Abuja, Nigeria. Abuja lies at latitude 9.07°N and longitude 7.48°E, and at an elevation of 840 m (2760 ft) above sea level. Abuja has two distinct seasons: The rainy season that lasts from April to October with rainfall ranging from 305 to 762 mm (12 to 30 in) and temperatures rising up to 40°C in May; and the dry season that lasts from November through March with dry winds lowering the temperature to as low as 12°C. It is bounded on the north by Kaduna state, on the west by Niger state, on the east and southeast by Nasarawa state, and on the southwest by Kogi state. The Federal Capital Territory (FCT) is divided into six area councils, namely, Abuja municipal, Gwagwalada, Abaji, Kuje, Bwari, and Kwali [26]. Samples were collected at Gwagwalada and Karu abattoirs. An average of 20 (Gwagwalada abattoir) and 50 (Karu) cows is slaughtered every day. The study population included cows and heifers slaughtered at the abattoir while the study unit comprised cows/heifers slaughtered on the day of visit.

Gwagwalada is one of the six area councils of Abuja, the FCT of Nigeria. It is about 45 km away from the Federal Capital City (FCC). The town is located between latitude 8°55’ and 9°00’N and longitudinal 7°00 and 7°05’E and lies in the downstream of River Usuma. It is bounded to Kogi state by the west [26]. Karu abattoir is located within Abuja municipal area council, which is also called FCC.

### Sample size calculation

The sample size was determined using the formula by Thrustfield [27].


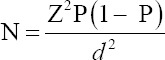


Where, N = sample size

Z= 1.96 standard normal value for desired confidence (normal distribution table)

P= prevalence rate from previous study

d = allowable error (5%)

Gimba [28] reported a prevalence of 6.60. Therefore:


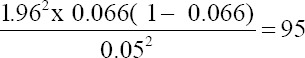


To minimize error and increase precision, 108 samples were used for this study.

### Experimental animals, clinical examination, and blood sample collection

A total of 108 cows and heifers were sampled. Animals showing signs such as emaciation, weakness, or other signs suggestive of trypanosomosis such as anemia, alopecia, or lymphadenopathy at point of slaughter were selected and examined. The history of the animals was taken to ascertain their source and general husbandry practice. Body condition of the study animals was scored based on the criteria set by Nicholson [29]. The body score was determined using a scale of 1 for very thin to 5 for fat animals. The age of the animals was estimated during the process of sampling based on the criteria set by Lasisi [30]. Five milliliters of blood was collected at slaughter and placed into EDTA bottle for parasitological examination. All blood samples were numbered 1-108 for identification and compared with the result of the study on reproductive abnormalities.

### Parasitological diagnosis and packed cell volume (PCV) evaluation

The 5 mL of blood collected in the EDTA bottle was subjected to diagnostic techniques of the standard trypanosome detection methods [31]; wet film, thin film, thick film, and concentration techniques, hematocrit centrifugation techniques [32], and buffy coat method [33]. Furthermore, the PCV of all animals was recorded using the microhematocrit method.

### Macroscopic examination

After slaughter, the reproductive organs were removed, closely inspected, and palpated for any gross pathological lesions as described by Assay [34] and Agrawal [35]. Thereafter, the genital organs were numbered, put in separate plastic bags, and taken to the laboratory on ice as described by Hatipoglu [36]. In the laboratory, the lesions were examined for distribution, texture, consistency, shape, size, and color. The tubular parts of the reproductive tract were dissected longitudinally and examined. The vagina was the first part of the tract to be opened and examined. The cervix was dissected from os externum to os internum. The body of the uterus, left and right uterine horns, and fallopian tubes was opened by midline incision and examined. The ovaries were examined externally and internally and all the findings were recorded [37]. Size of Graafian follicles and corpus luteum was measured by Vernier caliper [36].

### Microscopic examination

Pieces of the organs were fixed in Bouin solution and taken to the laboratory for histopathological processing. The organs were cut in slabs of about 0.5 cm thick transversely and transferred into varying degrees of alcohol. The fixed tissues were embedded in paraffin, sectioned at 5.0 mm thickness, and stained with hematoxylin and eosin [36].

### Statistical analysis

Results were analyzed using SPSS version 11.0. (SPSS Inc., Chicago, USA). Turkey’s test was used to compare data of PCV, age, and body score of the infected and non-infected animals. Chi-square test was used to test for associations among the prevalent trypanosome species. Chi-square test was used to determine presence of dependency between different variables and pathological abnormalities of reproductive organs. Significant level was set at p<0.05.

## Results

### Parasitological findings

The survey showed that the overall prevalence of trypanosomosis in the study sites was 5.55%. At Karu abattoir, 35 cattle were sampled and 2 (5.71%) were positive for trypanosomosis. In Gwagwalada abattoir, 73 cattle were sampled and 4 (5.48%) were positive for trypanosomosis. The proportion of trypanosome species was 66.67% (4/6) *T*. *vivax* and 33.33% (2/6) *T*. *congolense* (Table-1).

**Table-1:** Microscopic examination result.

Location	No. of animals sampled	No. of positive	Prevalence (%)	Species of trypanosomes			

				*T. vivax*	*T. congolense*	*T. brucei*	Mixed
Gwagwalada abattoir	73	4	5.48	3	1	0	0
Karu abattoir	35	2	5.71	1	1	0	0
Total	108	6	5.55	4	2	0	0

*T. vivax*=*Trypanosoma vivax*, *T. congolense*=*Trypanosoma congolense*, *T. brucei*=*Trypanosoma brucei*

### Prevalence of trypanosomosis according to age and body condition

A higher prevalence of trypanosomosis was observed in 2-5 years old than >5 years animals (Table-2). However, the variation in prevalence between the different age groups was not statistically significant (p>0.05). With respect to body condition score, the prevalence was 0%, 7.4%, and 13.3% in medium, poor, and very poor body condition, respectively, without a significant variation (p>0.05) between them.

**Table-2:** Trypanosome prevalence in relation to age and body conditions of the cattle.

Parameter	Total examined (n=108)	Positive cases (n=6)	p-value
Age			
2-5 years	43 (39.8)	3 (7.0)	0.600
>5 years	65 (60.2)	3 (4.6)	
Body condition			
Medium	39 (36.1)	0 (0)	0.112
Poor	54 (50.0)	4(7.4)	
Very poor	15 (13.9)	2 (13.3)	

### PCV

The range of the PCV values of animals examined for trypanosome infection was 10%-35%. The results showed that the mean PCV value for the parasitemic cattle was 22.83% while the mean PCV value for the aparasitemic cattle was 35.02% and the difference was highly significant, p<0.05 (Table-3). Cattle having PCV <24% (anemic) were 10 in number (9.26%) while the cattle having PCV ≥24% (non-anemic) were 98 in number (90.74%) and the differences were also significant, p<0.05 (Table-4). Of the 9.26% anemic cattle, 3.7% (4/108) were trypanosome-infected animals. More animals 5.6% (6/108) had anemia (PCV <24) without having trypanosome infection. Some animals 1.9% (2/108) were infected by trypanosome but their PCV was found normal (Table-4).

**Table-3:** Comparison of mean PCV between infected and non-infected cattle.

Condition	No. of examined	Mean PCV	SEM	t-test	p-value
Parasitemic	6	22.83	1.22	4.42	0.00
Non- parasitemic	102	35.02	0.67	
Total	108		35.16	1.08	

**Table-4:** Anemia proportion from trypanosome infected and non-infected cattle.

Trypanosome	Anemia	Frequency	Percent
Non-infected	Negative	96	88.9
	Positive	6	5.6
Infected	Negative	2	1.9
	Positive	4	3.7

### Uterine lesions

**Figure-1:**
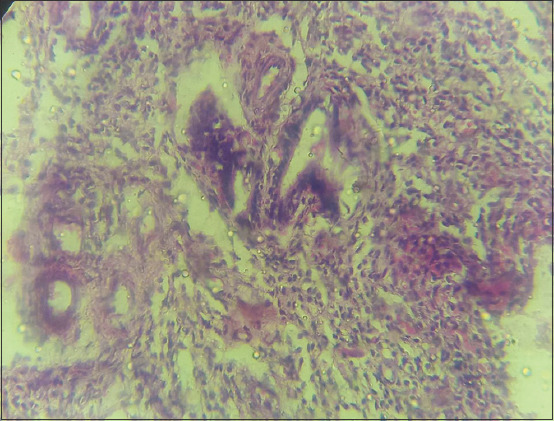
Photomicrograph of the uterus of cattle naturally infected with *Trypanosoma vivax*. Note the diffuse mononuclear cell infiltration in the endometrium. Hematoxylin and eosin stain×460.

**Figure-2:**
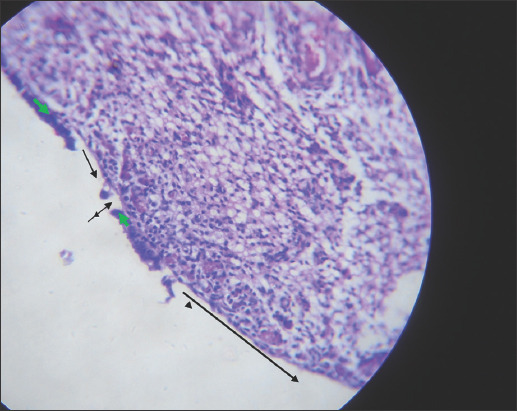
Photomicrograph of the uterus of cattle naturally infected with *Trypanosoma vivax*. Note sloughing of epithelium (black arrows) and necrosis of epithelium (green arrows). Hematoxylin and eosin stain×160.

**Figure-3:**
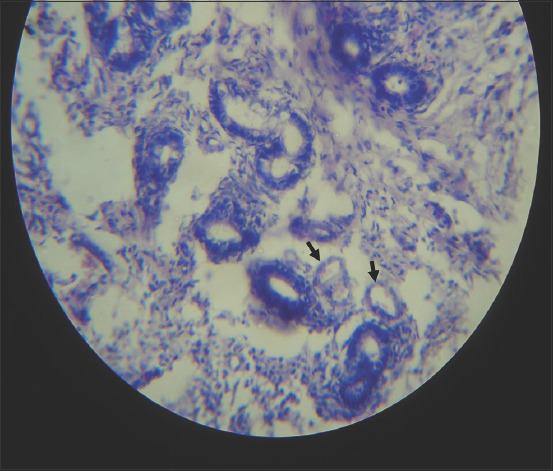
Photomicrograph of the uterus of cattle naturally infected with *Trypanosoma vivax* showing glandular endometrial atrophy (black arrows). Hematoxylin and eosin stain×160.

**Figure-4:**
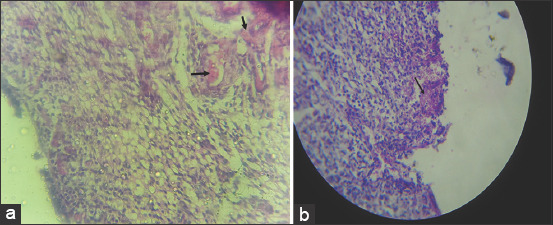
(a) Photomicrograph of the uterus of cattle naturally infected with *Trypanosoma vivax* showing areas of vascular congestion (black arrows). Hematoxylin and eosin stain×460. (b) Photomicrograph of the uterus of cattle naturally infected with *T. vivax* showing areas of vascular congestion (black arrow). Hematoxylin and eosin stain×160.

The uterine abnormalities observed in the infected animals are endometritis and mucometra. Histologically, the uterine lesion was characterized by mononuclear cell infiltration (Figure-1), sloughing of the endometrial epithelium (Figure-2), periglandular cell infiltration, necrosis of mucosal epithelium (Figure-2), and glandular endometrial atrophy (Figure-3). Similarly, vascular congestion of the endometrium was observed (Figure-4).

### Cervical and vaginal lesions

**Figure-5:**
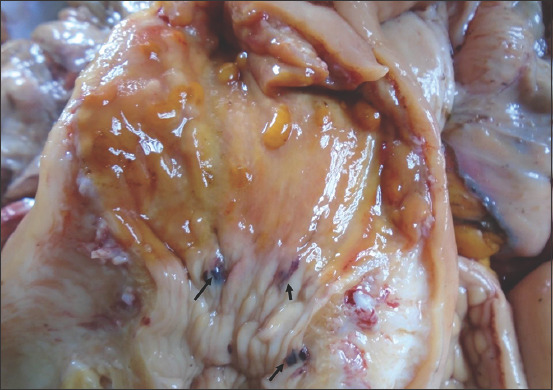
Cervix of cattle naturally infected with *Trypanosoma congolense* showing focal submucosal hemorrhages on the cervical mucosa (black arrows)×2.5.

The cervix of one animal positive for trypanosomes showed areas of focal submucosal ecchymotic hemorrhage, 3-5 mm in size, on the cervical mucosa (Figure-5). Vagina of positive samples did not show any significant lesion. Vaginal epithelium appeared normal with well-developed stratified squamous epithelium but congested blood vessels.

### Ovarian and oviductal abnormalities

**Figure-6:**
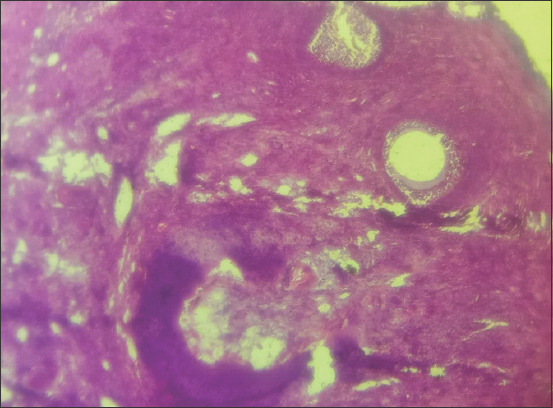
Photomicrograph of the ovary of cattle naturally infected with *Trypanosoma vivax*. Note the paucity of primordial follicles. Hematoxylin and eosin stain×460.

**Figure-7:**
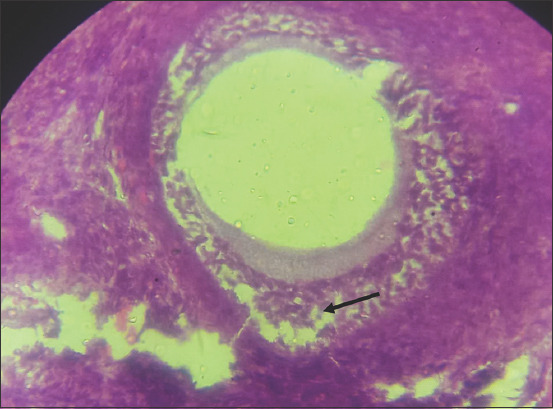
Photomicrograph of the ovary of cattle naturally infected with *Trypanosoma vivax*. Note the disorganization (degeneration) of the layers of granulosa cells (arrow). Hematoxylin and eosin stain×920.

**Figure-8:**
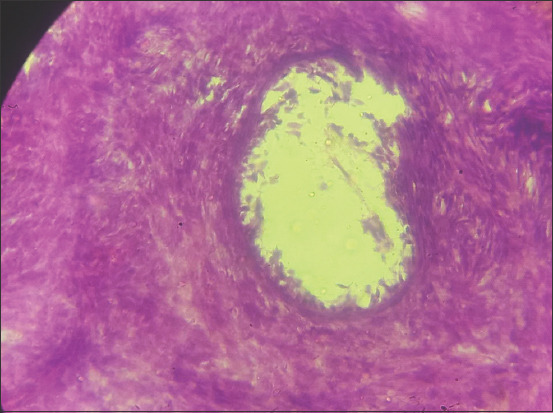
Photomicrograph of the ovary of cattle naturally infected with *Trypanosoma vivax* showing atrophy of granulosa and theca cells. Hematoxylin and eosin stain×920.

The ovaries of three animals that were positive for trypanosomes showed increased numbers of degenerating tertiary follicles and absence of corpora lutea (Figures-6 and 7). There was also atrophy of the granulosa and theca cells (Figure-8). Thickening of the oviduct (Salpingitis) was found in both oviducts of two samples.

## Discussion

The overall abattoir prevalence of trypanosomosis (5.55%) reported in this study is similar to the value of 5.0% reported by Ezebuiro *et al*. [5] in cattle slaughtered at Kaduna abattoir. Adama *et al*. [38] also reported a similar rate (6.3%) in cattle within Niger state. It is, however, lower than the prevalence reported by Majekodunmi *et al*. [39] who reported a rate of 46.8% in Plateau state and Hassan *et al*. [33] who recorded a prevalence rate of 53.33% in Lafia abattoir. Majekodunmi *et al*. [39] used molecular diagnostic techniques (polymerase chain reaction, gel electrophoresis), which permit precise identification of the parasite to species level. The lower prevalence observed in this study compared to Majekodunmi *et al*. [39] could be due to inadequacy of the microscopic and concentration detection methods and intermittent parasitemia [40]. Lower prevalence could also be as a result of parasite control program practiced in the area.

The infection rate for *T*. *vivax* (66.67%) is higher than that of the other trypanosome species (*T. congolense*, 33.33%) in the current study. This is in agreement with the previous results of Ezebuiro *et al*. [5] (60%) and Hassan *et al*. [33] (43.05%). This could be because *T. vivax* is transmitted by other biting flies apart from *Glossina*. However, Majekodunmi *et al*. [39] (27.7%) and Samdi *et al*. [41] (50%) reported a higher *T*. *congolense* infection rate. This disparity suggests that all the three principal pathogenic trypanosomes of ruminants are important cause of the disease in Nigeria [7].

Equal chance of exposure to the trypanosome parasite may have affected the rate of infection among different age groups and body condition in this study. This finding is in agreement with previous works [5,42-44].

In this study, the overall prevalence of 9.26% for anemia was significantly higher in animals positive for trypanosomes (4/6, 66.66%) than in non-infected animals (6/102, 5.88%) when both were compared (p<0.05). This result agrees with previous reports by Abenga [6], Biyazen *et al*. [42] and Mihret and Mamo [45]. Of the 9.26% anemic cattle, 3.7% (4/108) was trypanosome-infected animals. More animals, 5.6% (6/108) had anemia (PCV <24) without having trypanosome infection. This implies that other factors such as infestation with gastrointestinal parasites, nutritional deficiencies, and other vector-borne diseases could have been responsible for the reduction in the PCV of animals or due to the inadequacy of the methods used. The few animals that were positive for trypanosomes, who’s PCV was more than 24% suggest that the infection was recent [46].

The mean PCV of the parasitemic (22.83±1.22) and non-parasitemic (35.02±0.67) cattle in the current study indicated a significant difference. The low PCV of the infected cattle which is indicative of anemia, is in agreement with previous findings [6,44,47-49]. Low PCV value may not solely be due to trypanosomosis. However, the difference in mean PCV value between parasitemic and aparasitemic animals indicates that trypanosomosis is involved in reducing the PCV values in the infected animals.

In this study, the uterine pathological lesions were similar to reports in heifers infected with *T*. *congolense* [8], in pregnant ewes infected with *T*. *vivax* [18], and in goats infected with *T*. *brucei* [50]. However, it is in contrast with previous studies, reported that there were no gross lesions in the uterus of *T*. *evansi* infected Yankasa ewes [51] and *T*. *congolense* infected West African Dwarf (WAD) goats [52]. Abubakar *et al*. [52] reported that WAD is trypanotolerant. *T. evansi* is not as virulent as *T. vivax* and *T. congolense* in ruminants [53]. In the present study, the lesions may be compounded by other organisms such as *Arcanobacterium pyogenes*, *Escherichia coli*, or *Streptococcus pyogenes* [54].

Histopathologically, the uterine lesions observed are in consonance with reports of horses naturally infected with *Τrypanosoma equiperdum* [15], heifers infected with *T*. *congolense* [8], and goats infected with *T*. *brucei* [50]. These inflammatory and degenerative changes may cause infertility, embryonic death, and abortion in pregnant cows, as stated by Ogwu and Njoku [8], Leigh *et al*. [50], Jones *et al*. [55], Mcentee [56].

In the current study, two cases positive for trypanosomes had mucometra. This is in contrast with reports by all authors who experimentally infected various animal species with trypanosomes [8,18,50-52,57]. This result implies that some factors or organisms may be responsible for the mucometra not trypanosomes.

The ovarian lesions observed in this study are similar to reports by Ogwu and Njokwu [8] who reported follicular cystic degeneration in heifers experimentally infected with *T. vivax* and *T*. *congolense*, respectively. Rodrigues *et al*. [14] also reported similar findings in goats experimentally infected with *T. vivax* from the Brazilian semi-arid region. The other *Trypanosoma*-positive cases had relatively normal ovarian histology. This could be due to the duration/stage of infection and the ability of these animals to restore their fertility following natural infection. Due to the trypanotolerant nature of WAD ewes, Abubakar *et al*. [52] also reported no lesion on the ovary of WAD ewe experimentally infected with *T*. *congolense*. Ogwu *et al*. [58] and Llewelyn *et al*. [59] reported anestrus in cows experimentally infected with *T. vivax* and *T. congolense*, respectively. Anestrus may have occurred in some trypanosome-positive animals which had smooth ovaries without tertiary follicles or corpora lutea. Adenowo *et al*. [13] and Mutayoba *et al*. [60] also reported severe fibrosis and degeneration of ovarian stroma, and atretic follicles.

Cervicitis was observed in one sample of the six trypanosome-infected animals. This is in agreement with the report by Bawa *et al*. [61]. There were no significant observable lesions in the vagina of trypanosome positive samples. This is in contrast with the report by Ogwu and Njoku [8], in heifers infected with *T*. *congolense* who reported mucosal desquamation and mononuclear cell infiltration.

## Conclusion

This study indicated that trypanosomosis is an important disease and a potential threat to the productivity and health of cattle in Abuja and its environment. The major species of trypanosomes in the study area were *T*. *vivax* and *T*. *congolense*. Trypanosomosis is involved in the reduction of PCV of infected animals. More young animals (≤5 years of age) constitute the number of animals sampled. This indicates that trypanosomosis and other debilitating diseases negatively affect the productivity of animals in the study area. The manifestation of genital lesions in trypanosomosis induced by natural infection is dependent on the stage and severity of *Trypanosoma* infection and is different from findings in experimental infections.

## Authors’ Contributions

KOA: Conducted the study and drafted the manuscript. DO: Supervised the project and reviewed the manuscript. LA: Conceived the original idea, supervised the project, and reviewed the manuscript. CEO and JII: Managed the analysis. NAS: Participated in gross and histopathological analysis of the samples. All authors read and approved the final manuscript.
